# Why do dogs look back at the human in an impossible task? Looking back behaviour may be over-interpreted

**DOI:** 10.1007/s10071-020-01345-8

**Published:** 2020-02-23

**Authors:** Martina Lazzaroni, Sarah Marshall-Pescini, Helena Manzenreiter, Sarah Gosch, Lucy Přibilová, Larissa Darc, Jim McGetrick, Friederike Range

**Affiliations:** 1 Domestication Lab, Konrad Lorenz Institute of Ethology, University of Veterinary Medicine, Vienna, Austria; 2 Comparative Cognition, Messerli Research Institute, University of Veterinary Medicine, Vienna, Austria

**Keywords:** Looking back, Impossible task, Persistence, Free-ranging dogs

## Abstract

The impossible task paradigm has been extensively used to study the looking back behaviour in dogs. This behaviour is commonly considered a social problem-solving strategy: dogs facing an unsolvable task, soon give up and look back at the experimenter to ask for help. We aimed to test if the looking back in an impossible task does indeed represent a social problem-solving strategy. We used a modified version of the classic impossible task, in which the subjects simultaneously faced three possible and one impossible trials. Additionally, subjects were tested in four different conditions: social condition (with an unknown experimenter); asocial condition (subject alone); 'dummy' human condition (with a ‘dummy’ human); object condition (with a big sheet of cardboard). Finally, we compared two populations of dogs differing in their experience of receiving help from humans: 20 pet dogs tested in their houses and 31 free-ranging dogs tested in Morocco. We found that the pet dogs and free-ranging dogs had similar persistence in interacting with the impossible task in all conditions. Moreover, subjects looked back with similar latencies at the human, at the dummy human and at the object. Overall, pet dogs looked back longer at the human than free-ranging dogs. This could be interpreted as pet dogs being more attracted to humans and/or having a stronger association between humans and food than free-ranging dogs. Concluding, the looking back in an impossible task does not represent a problem-solving strategy. This behaviour seems rather linked to the subject’s persistence, to the salience of the stimuli presented, and potentially to the past reinforcement history.

**Electronic supplementary material:**

The online version of this article (10.1007/s10071-020-01345-8) contains supplementary material, which is available to authorized users.

## Introduction

Humans look at each other in many situations and often directly at each other’s faces to collect information about others’ intentions and mental states. This ability allows for a complex social communication (Bruce and Young 1998). Interestingly, dogs also gaze at their human partners in many different situations, which is often interpreted as serving similar functions as in humans (Hare and Tomasello 2005). This propensity to look at us has been suggested to have evolved during the domestication process enabling the close dog–human communication characterizing our relationship (Hare and Tomasello 2005; Hare et al. 2002). In their seminal study, Miklósi et al. (2003) compared the looking behaviour towards humans in an impossible task paradigm between dogs and human-socialized wolves, which likely resemble dogs’ closest non-domesticated ancestors (Lindblad-Toh et al. 2005). In this paradigm, the subject is faced with a series of possible trials, in which the animal can independently solve the problem to obtain a food reward. Following the possible trials, an impossible trial is presented, which is identical to the prior ones, but it is no longer solvable. When facing the impossible trial, dogs looked back at the human sooner and for longer than the wolves, which instead persisted in the attempt to reach the reward. Since the two groups showed similar food motivations, the authors suggested that dogs “were bound to a lesser degree to the ‘attracting’ effects of the food”, being instead more attracted by the human and thus facilitating dog–human communication (Miklósi et al. 2003).

Since then, the impossible task paradigm has been extensively used to study the looking back behaviour in many different contexts: domestication (Miklósi et al. 2003; Marshall-Pescini et al. 2017; Smith and Litchfield 2013); training (Marshall-Pescini et al. 2009, 2016; D’Aniello et al. 2015); breed differences (Konno et al. 2016; Brodd 2014; Marshall-Pescini et al. 2016); genetic bases and heritability (Hori et al. 2013; Persson et al. 2015); aging (Passalacqua et al. 2011; Brodd 2014); effect of experience (D’Aniello and Scandurra 2016; Marshall-Pescini et al. 2017); reputation (Piotti et al. 2017) and comprehension of the other’s attentional stance (Marshall-Pescini et al. 2013). In quite a number of these studies (Piotti et al. 2017; Brodd 2014; Passalacqua et al. 2011; Hori et al. 2013; Konno et al. 2016; Marshall-Pescini et al. 2016; Persson et al. 2015; D’Aniello and Scandurra 2016), the authors hypothesised that the looking back was not only determined by ‘human’s attractiveness’ to dogs, but also that dogs might use this looking behaviour as an alternative problem-solving strategy (i.e. asking the human for help). It has been investigated whether dogs assess the skilfulness of the human, enlisting her help specifically (Piotti et al. 2017), and whether primitive breeds (wolf-like) were less prone to use this behaviour than other breeds (Passalacqua et al. 2011). Other studies have shown that experience of living in close contact with humans strongly affects some aspects of the looking back behaviour. It has been found that adult dogs looked longer at the experimenter than juvenile dogs (Passalacqua et al. 2011; Brodd 2014) and that pet dogs looked back longer than dogs living in kennels and thus had far less experiences with human interactions (D’Aniello and Scandurra 2016).

The results of these studies using the impossible task paradigm suggest a selection for a tendency to look at the human in dogs during the domestication process, which would facilitate the development of complex socio-communicative skills given the right experience. This could include requesting help in specific situations and would be in line with the hypothesis that both domestication and subjects’ experience play a role in the emergence of dogs’ socio-cognitive skills (Reid 2009; Miklósi and Topál 2013).

Whether this looking behaviour in these situations indeed functions as a communicative signal that can be interpreted as a request for help has never been properly tested. Indeed, two studies have recently questioned the interpretation of the looking back behaviour in the impossible task paradigm (Marshall-Pescini et al. 2017; Udell 2015). Both studies suggested that the shorter latency in the looking back behaviour of dogs in comparison with wolves might be due to a lower persistence in dogs compared to wolves in their interaction with the object. Udell et al. (2015) suggested that such lower persistence in dogs may be determined by a degree of social inhibition enforced by humans on dogs in their everyday life. Marshall-Pescini et al. (2017) found that indeed subjects' persistence (i.e. duration of interaction with the object during the impossible trial) emerged as the best explanatory variable to account for differences between wolves and dogs, suggesting that their differential looking behaviour did not reflect different problem-solving strategies or attraction to the human face. Moreover, contrary to what was expected, the authors did not find any difference in the latency to look back, among populations of dogs with different experiences of human help (i.e. free-ranging dogs, captive pack-living dogs and pet dogs). The authors suggested that the looking back behaviour may be the direct consequence of giving up and then turning to the most salient stimulus (the human) in the environment rather than a ‘social/communicative’ strategy aimed at solving the problem (Hall 2017). However, based on these results alone it could still be argued that dogs may voluntarily give up the task sooner to ask for help, while wolves try to solve the task independently from the human (Udell 2015; Marshall-Pescini et al. 2017).

Another problem with the task that has not been considered so far is in the procedure itself: in the majority of studies the experimenter manipulates the apparatus, refilling it with the food and presenting the possible trials consecutively one after the other, ending with the impossible trial (but see Persson et al. 2015; Brodd 2014). This procedure might influence subjects’ perception of the experimenter’s role, leading the dogs to look back at the experimenter just because they associate the experimenter with refilling the empty apparatus with food (Horn et al. 2009). Moreover, in some studies, subjects were kept on the leash while tested and the coercion to face the trials might have induced subjects’ looking back at the handler (Smith and Litchfield 2013).

The aim of the current study was to test if the looking back behaviour in an impossible task represents a social problem-solving strategy and if experience with human helping affects the occurrence of this behaviour. We used a modified version of the impossible task, in which the subjects simultaneously faced three possible and one impossible apparatuses, but never observed a person interacting with the apparatus and handling the rewards. Furthermore, the subjects were tested in four different conditions (one social condition and three control conditions): social condition (the experimenter was present); asocial condition (the subject was alone); ‘dummy’ human condition (a cardboard shaped as a human and with a human painted on it was present); object condition (a non-human-shaped cardboard was present). Furthermore, we compared two populations of dogs differing in their experiences with humans: pet dogs (Pd), which have ample experience of human’s help, and a free-ranging dog (FRd) population, which have no experience of humans helping, but are well socialized to humans and are likely to have associated humans with food, given their daily experiences. Indeed, our study population of free-ranging dogs consists of scavengers whose main source of food is represented by human waste and that predominately obtain food autonomously. Although they are occasionally directly fed by the human population, in two 6-month-long field seasons, we never observed any form of ‘helping’ whereby a person would aid a dog in obtaining food. The closest observation of this type of interaction occurred on only a few occasions, in which we observed dogs obtaining food from a man who was rummaging in a bin (inaccessible to dogs) and accidentally dropped some food, which the dogs quickly obtained.

Our predictions (see Table 1) are based on the main hypotheses of whether the looking back functions as a problem-solving strategy: (1) looking back is an acquired problem-solving strategy, which might be either favoured by an effect of domestication on dogs’ overall tendency to look at the humans (1a) or only be determined by subjects’ experience (1b). (2) Alternatively, looking back behaviour is either a consequence of a reduced persistence resulting in dogs ceasing the interaction with an apparatus and then looking at the most salient stimulus in the environment—usually the human (2a) or dogs looking at the human because of selection for this behaviour, but it does not function as a problem-solving strategy, but rather occurs in all situations (2b). Aside from the usual behavioural measures (persistence in manipulating the task and latency, frequency and duration of looking behaviour towards the human), we also analysed the dog’s tail position and wagging behaviour. Since the behaviour of free-ranging dogs towards people can vary widely across populations, we used tail wagging as a proxy for positive arousal and assessed it in both the pet and free-ranging population.

**Table 1:** List of the hypotheses and predictions

	Predictions			
	Persistence^a^	Latency and frequency of looking back when manipulating the impossible bowl	Overall duration of looking back	
1. Looking back is a problem-solving strategy				
1a. Domestication has selected in dogs the tendency to look at the human, which favoured the development of looking back as a problem-solving strategy	Pd should be less persistent in the social condition than in the control conditions and less persistent than FRd in the social condition	All subjects should look back sooner and more frequently in the social condition than in the control conditions but Pd should look back more frequently after attempting the impossible bowl than the possible bowls	All subjects should look longer at the human than at the objects, but Pd should look longer at the human than FRd	
1b. Domestication did not have a selective effect on dogs’ tendency to look at the human but looking back represents an acquired problem-solving strategy	Pd should be less persistent in the social condition than in the control conditions and less persistent than FRd in the social condition	Pd should look back sooner and more frequently in the social condition than in the control conditions and more frequently after attempting the impossible bowl than the possible bowls. FRd should look back with similar latency and frequency in all conditions	Pd should look longer at the human than at the objects and should look longer at the human than do FRd. FRd may look longer at the human than at the object due to the higher saliency of the human or they may not look at the human at all	
2. Looking back is not a problem-solving strategy				
2a. Looking back is only driven by the dogs giving up and then looking at the most salient object	Subjects should be equally persistent in all conditions	Subjects should look back with similar latency and frequency in all conditions	Subjects should look longer at the human than at the objects and longer at the human shape than at the cardboard. Pd should look longer at the experimenter than FRd due to their stronger relationship with the humans	
	No differences between pet dogs and free-ranging dogs			
2b. Domestication has selected in dogs the tendency to look at the human face independently of the situation	Subjects should be equally persistent in all conditions	Subjects should look with a shorter latency and more frequently at the human than at the objects but with similar frequency after attempting the impossible bowl or the possible ones	Subjects should look longer at the human than at the objects and longer at the human shape than at the cardboard	
	Overall no differences between pet dogs and free-ranging dogs			

*Pd* pet dogs, *FRd* free-ranging dogs

^a^Persistence refers to the total duration of interaction with the impossible bowl

## Materials and methods

## Ethical statement

Ethical approval for this study was obtained from the ‘Ethik und Tierschutzkommission’ of the University of Veterinary Medicine Vienna (Protocol number ETK-16/09/2017, ETK-20/09/2017). Informed consent was obtained by all owners of the pet dogs. The authorization to test the free-ranging dogs was provided by the municipality of Taghazout (Morocco).

### Subjects

*Pet dogs (Pd).* Mixed-breed pet dogs were tested in private homes in Austria. The subjects were recruited from both the Clever Dog Lab database and via social media. A total of 20 pet dogs were tested (13 F, 7 M; mean age in years: 6.3 ± 0.6 SE).

*Free-ranging dogs (FRd).* Free-ranging dogs were tested in their natural environment in the municipality of Taghazout, Agadir, Morocco. The experimenters (ML, LD and RM) travelled by car to look for solitary dogs (solitary dogs were chosen to avoid interference by conspecifics). Only adult dogs (appearing to be over 1 year of age) were tested. A total of 62 dogs performed at least one test condition. A total of 31 dogs were excluded from the analyses, because other dogs interfered during the test. Hence, a total of 31 free-ranging dogs (12 F, 19 M) were included in the analyses. The tested free-ranging dogs were village dogs living around human settlements. They were well socialized with humans and had daily experience of humans near their food sources (mainly garbage). Many of them also experienced receiving food directly from humans. However, while the dogs might relate humans to food, they did not receive help from humans in obtaining food.

### Apparatus

The apparatus consisted of a wooden board (length: 1 m, width: 0.5 m) with four overturned transparent and perforated containers baited with three types of food simultaneously (dry food, sausage and cheese). Three out of the four containers could be moved (possible bowls), whereas the fourth one was attached to the board (impossible bowl). To avoid habituation to the apparatus, we used different shapes of containers for each condition (bottom of a rigid plastic bottle, top of a rigid plastic bottle, or Tupperware box) that were counterbalanced across dogs and conditions (e.g. a subject experienced the Tupperware box as the container in condition 1, and the rigid plastic bottle top as the container in condition 2, whereas another subject might have experienced the rigid plastic bottle bottom in their condition 1 etc.). The objects were chosen to be at least somewhat familiar to the free-ranging dogs.

### Testing procedure

**Fig. 1:**
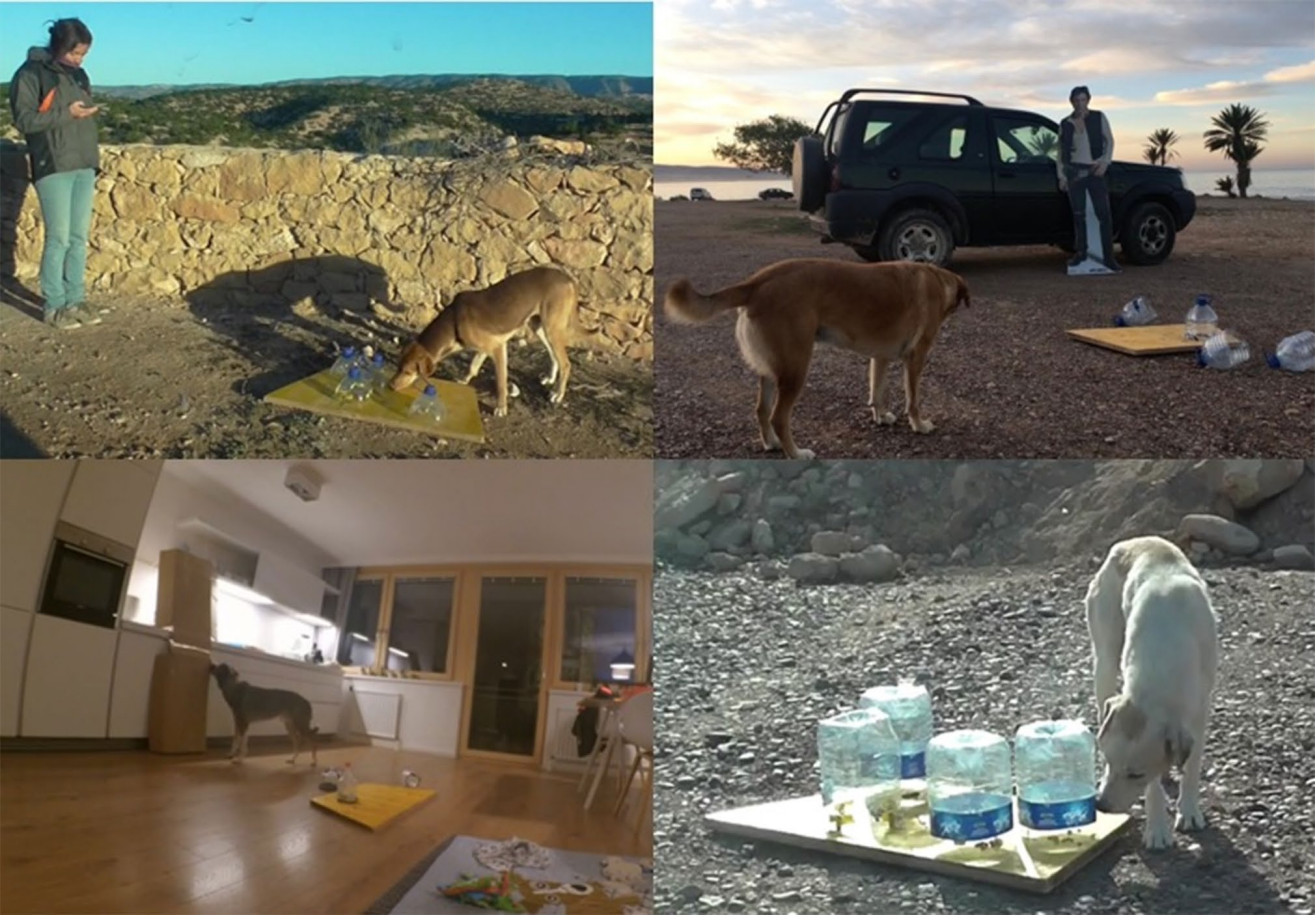
The four conditions presented to the subjects: social (a human present); dummy (a dummy human present); object (a big cardboard present); alone (the dog is alone). Three pictures of free-ranging dogs and one of a pet dog are shown

All pet dogs were tested in all four test conditions: (1) social; (2) dummy; (3) object; (4) alone (80 tests in total with Pd). 14 of the free-ranging dogs were tested in the social and alone conditions, while 17 naïve dogs were tested only in the ‘dummy’ human condition (45 tests in total with FRd). Depending on the test condition (social, dummy, object), the experimenter, a ‘dummy’ human (Han Solo figure, width: 59 cm, height: 186 cm) or a ‘dummy’ object (width: 64 cm, height: 188 cm) were standing at 1.5 m behind the apparatus. Both the objects and the experimenter were unknown to the dogs (see Fig. 1). Where we were able to test the same dogs twice the conditions were counterbalanced across dogs.

*Pet dogs (Pd).* Subjects were tested in their owner’s homes in Vienna. The animals were initially moved to a different room before the apparatus was placed in the testing room. Once the apparatus, cameras and where applicable, the human, the human-shaped cardboard and the object were in place, the dog was led into the room. For the social condition, the experimenter stood at a distance of 1.5 m from the apparatus looking at her phone during the entire test, while the owner was waiting in a separate room. For the other conditions, both the owner and the experimenter left the house after giving a ‘goodbye’ signal to the dog according to the usual routine of the specific dog–human dyad. All tests were recorded using two cameras with one camera being remotely controlled to observe the subject in the three conditions in which it was left alone in the room.

*Free-ranging dogs (FRd).* Free-ranging dogs were tested in the streets and on the beaches of Taghazout. Once a subject was located alone, the experimenter placed the apparatus on the ground, taking care not to be seen by the subject. The experimenter then stood at 1.5 m behind/next to the apparatus (for the social condition) or hid in the car (control conditions). A second experimenter went to the dog, petted it for a few seconds and then walked towards the apparatus making sure that the dog followed. The experimenter did not show the apparatus to the dog, but simply walked past it and then got into the car. The test started when the dog approached the apparatus. All tests were filmed from the car or from the experimenter standing in front the apparatus (social condition).

The tests started when the dog approached the apparatus (i.e. when they were within 10 cm of the apparatus) and ended if the subject stopped interacting with the apparatus (sniffing it or manipulating it) for 5 min. Thus, the whole test duration was not fixed, but determined by the behaviour of the subject (Online Resource 2). We tested pet dogs and free-ranging dogs in different environments (indoor and outdoor), because the common and most important feature was that both groups were tested in their most familiar environment, where it was assumed that they would feel most comfortable.

### Analyses

All the videos were coded using the software Solomon coder (developed by András Péter, Dept. of Ethology, Budapest, www.solomoncoder.com). See Table 2 for definitions of the coded behaviours.

**Table 2:** Detailed description of the coded behaviours

Persistence	The subject sniffs and/or manipulates, either with the paw or the nose, the impossible bowl (duration^a^)
Looking back	The subject turns/lifts its head towards any part of the experimenter’s body, the ‘dummy’ human or the object (duration^a^, latency^b^, frequency^c^)
Looking up	The subject raises its head up from the ground soon after (max 2 s) interacting with the bowl (frequency)
Emotional arousal	Tail wagging: the subject moves rapidly the tail from side to side. The tail may be perpendicular to or below the plane of the back (duration^a^)

^a^The duration of persistence, looking back and tail wagging is collected for the whole test duration

^b^The latency of looking back is the interval of time that elapses between the first time that the subject sniffs or touches the impossible bowl—once all the reachable food is eaten—to the first time the subject looks back

^c^The frequency of looking back is the number of times the subject looks back after interacting with the impossible or the possible bowls (it is counted only if it occurs within a two second frame from the end of the interaction with the bowl)

Inter-observer reliability was carried out between three observers, each coding 20% of the video data (Intra-class correlation coefficient: persistence ICC = 0.99, looking back frequency ICC = 0.81, looking back duration ICC = 0.82, looking back latency ICC = 0.96).

For statistical analyses, we used generalized linear models (GLM) and generalized linear mixed models (GLMM). All models were fitted in R (version 3.6.1; R Core Team 2019) using the functions lm (R package stats), lmer (R package lme4) (Bates et al. 2014), glmmTMB (Brooks et al. 2017) and coxme (Therneau 2019). Model residuals of Gaussian models were tested for normality and homogeneity using diagnostic plots. Where the initial model did not fit the assumption of normally distributed residuals ,(models P1, P2, P3, L2, L3) we applied the Box-Cox transformation method, using the package MASS (Venables and Ripley 2002), and the appropriate transformation was applied to the response variable to achieve normally distributed residuals (log transformation for models P1, P2, P3, L3 and square root transformation for model-L2) (Venables and Ripley 2002). However, we decided to show in the graphs the non-transformed data. Collinearity of predictors, assessed applying the function vif of the R package car (Fox et al. 2012), appeared not to be an issue (Quinn and Keough 2002). Overdispersion appeared not to be an issue (range of dispersion parameters 0.19–1.17) except for models DL2, DL3, W2, W3 where we applied a function kindly provided by Roger Mundry to correct SE, z-, and P values for individual predictors. We assessed model stability on the level of the estimated coefficients and standard deviations by excluding the levels of the random effects one at a time (Nieuwenhuis et al. 2012). Overall, all models except model-F1, model-F2 and model-F4 were of moderate or good stability (Online Resource 1). For models including more than one predictors, P values for the individual effects were based on likelihood ratio tests comparing the full model with the respective reduced models lacking the model predictors (R function ‘anova’) (Barr 2013).

Results were supplemented with Bayes factors, which were computed with the BayesFactor package (Morey and Rouder 2018) using the functions anovaBF and lmBF. For models DL1, DL2, DL3, W1, W2, W3 Bayes factors were manually calculated using the BIC approximation (Wagenmakers 2007). Whenever non-significant results were found using frequentist inference statistics, the null hypothesis cannot be rejected. Bayesian statistics allow a determination of whether the data provide stronger evidence for H1 or the null hypothesis (H0). The value of the Bayes factor (BF) indicates the number of times the data are more likely under the H1 hypothesis than under the H0 null hypothesis. A BF higher than one gives stronger support to the H1 hypothesis than the H0 hypothesis, while a BF smaller than one is in support of the H0 hypothesis rather than the H1 hypothesis. Conventionally, a BF > 3 can be interpreted as substantial evidence, whereas a BF > 10 is considered strong evidence (Rouder et al. 2018; Lee and Wagenmakers 2014). Plots were created in R using the package ggplot2 (Wickham 2009).

*Persistence.* The subjects that did not interact with the impossible bowl (Pd: object 1; FRd: social 1, alone 1) were excluded from the analyses of persistence, as persistence refers specifically to the duration of interacting with the impossible bowl. Two generalized linear mixed models (model-P1 for Pd and model-P2 for FRd) were run with persistence as the response variable, test order and condition (social, alone, object, dummy for Pd; social and alone for FRd) as explanatory factors and subject ID as random factor. To investigate differences in persistence between Pd and FRd in the presence of the human experimenter (social condition), a linear model (model-P3) was run with persistence as the response variable and group (Pd, FRd) as explanatory factor. The null models lacked the predictor condition for the comparison with model-P1 and model-P2. We calculated Bayes factors for condition in model-P1 and model-P2 and for group in model-P3.

*Latency of looking back.* In these analyses, we considered the latency to look back after attempting the impossible bowl once all the reachable food was eaten (see Table 2). The subjects that did not interact with the impossible bowl after the food was eaten were excluded from these analyses (1 Pd, 5 FRd). To investigate the differences in the latency of looking back between conditions (social, dummy and object) in Pd, we ran a Cox mixed-effects model (model-L1). A survival response variable was constructed using the Surv function (Therneau 2015), considering the latency to look back (or termination of the experiment) and whether this event occurred or not. Subject was included in the model as a random factor. Given that in the social condition all Pd looked back, to ensure model convergence we considered one subject in the social condition (with the longest latency to look back) as not having performed the behaviour. All FRd that finished the food and attempted the impossible bowl (ten social, nine dummy) looked back at the experimenter or at the dummy human, except one, which was excluded from the next analysis. To investigate the differences, in the latency to look back, between conditions in FRd, we ran a linear model (model-L2) with latency to look back as the response variable and condition (social, dummy) as the explanatory factor. To investigate differences in the latency to look back between Pd and FRd in the presence of the human experimenter, we ran a linear model (model-L3) with latency to look back as the response variable and group (Pd, FRd) as the explanatory factor. We calculated Bayes factors for condition in model-L1 and model-L2 and for group in model-L3, excluding the subjects that did not look back.

*Effect of condition and group on the frequency of looking back after attempting the impossible bowl.* The subjects that never attempted the impossible bowl were exclude from these analyses (Pd: object 1; FRd: dummy 3). To investigate the differences in the frequencies of looking back after attempting the impossible bowl between conditions in Pd and FRd, a generalized linear mixed model for Pd (model-F1) and a generalized linear model for FRd (model-F2) with a quasibinomial distribution were run with the occurrence of looking back (see Table 2) as the response variable, normalized by the total number of times the subject attempted the impossible bowl, condition (social, dummy, object for Pd; social and dummy for FRd) as the explanatory factor and subject as the random factor (only for model-F2). To investigate the differences in the frequencies of looking back at the experimenter after attempting the impossible bowl between pet dogs and free-ranging dogs, a binomial model with a quasibinomial distribution (model-F3) was run with the occurrence of looking back as the response variable, normalized by the total number of times the subject attempted the impossible bowl, and group as explanatory factor (this analysis was run only for the social condition). The null models lacked the predictor condition for the comparison with model-F1.

*Effect of group and the obtainability of food (possible vs. impossible bowl) on the frequency of looking back.* These analyses were run on the whole test duration, only for the social condition. We investigated the differences in the frequencies of looking back between Pd and FRd, after attempting either the possible or the impossible bowl. We ran a generalized linear mixed model (model-F4) with a binomial distribution with the occurrence of looking back (see Table 2) as the response variable, normalized by the total number of times the subject looked up (see Table 2) after attempting the bowl. The group, the attempted bowl (possible or impossible) and their interaction were included as explanatory factors. The null model lacked both predictors.

*Duration of looking back and emotional arousal.* These analyses were run on the whole test duration. All tested subjects were included in these analyses. To investigate whether the proportion of time individuals looked back or tail wagged at the experimenter differed between conditions, we ran two Generalized Linear Mixed Models for Pd (model-DL1 and model-W1) and two Generalised Linear Models for FRd (model-DL2 and model-W2) with beta error structure and logit link function. We included condition (social, object, dummy for Pd; social, dummy for FRd) as explanatory factor and subject as a random factor (only for model-DL1 and model-W1).

To investigate whether the proportion of time individuals looked back or tail wagged at the experimenter differed between Pd and FRd in the social condition, we ran two Generalised Linear Models (model-DL3 and model-W3) with beta error structure and logit link function. We included group (Pd, FRd) as an explanatory factor. For model-DL3, to account for possible more distractions in FRd than in Pd, which were tested outdoors, the response variable was the total time that the subjects looked at the experimenter divided by the total time the subjects looked up (see Table 2). For all the other models (model-DL1, model-W1, model-DL2, model-W2, model-W3) the response variable was the total time that the subjects looked or tail wagged divided by the total duration of the test.

The null models lacked the predictor condition for the comparison with model-DL1 and model-W1. We calculated Bayes factors for condition in model-DL1, model-W1, model-DL2, model-W2 and for group in model-DL3 and model-W3.

## Results

**Fig. 2:**
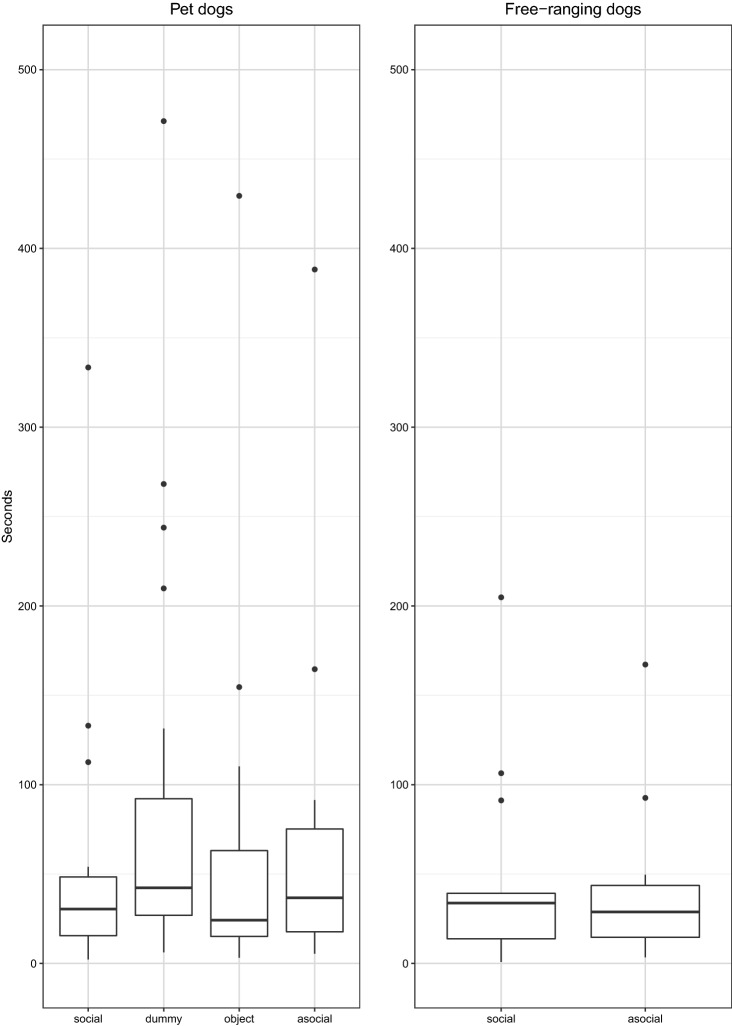
Persistence of interacting with the impossible bowl for pet dogs (*N* = 20) and free-ranging dogs (*N* = 14). One pet dog tested in the asocial condition, with a value of 806.2 s, is not shown in the graph

*Persistence of manipulating the impossible bowl*. Both the mixed model analysis and the Bayes factor analysis indicated that there was no effect of condition on persistence in Pd and FRd and there was no difference in persistence between Pd and FRd in the social condition (see Table 3; Fig. 2) (Online Resource 1). (See also Online Resource 3 reporting all raw data and Online Resource 4 reporting a summary of all data used in the analyses).

**Table 3:** Differences in persistence, summary of statistics

Differences in persistence	Model	Comparisons full-null model/tests	Bayes factor (support for H1)	Bayes factor (support for H0)
Across conditions in Pd	Model-P1	$\chi_{3}^{2}$ = 4.2,* p *= 0.24	0.14	6.93
Across conditions in FRd	Model-P2	$\chi_{1}^{2}$ = 0.43,* p* = 0.51	0.38	2.59
Between Pd and FRd (social condition)	Model-P3	*t* = 0.34, *p* = 0.73	0.35	2.89

*Pd* pet dogs, *FRd* free-ranging dogs

**Fig. 3:**
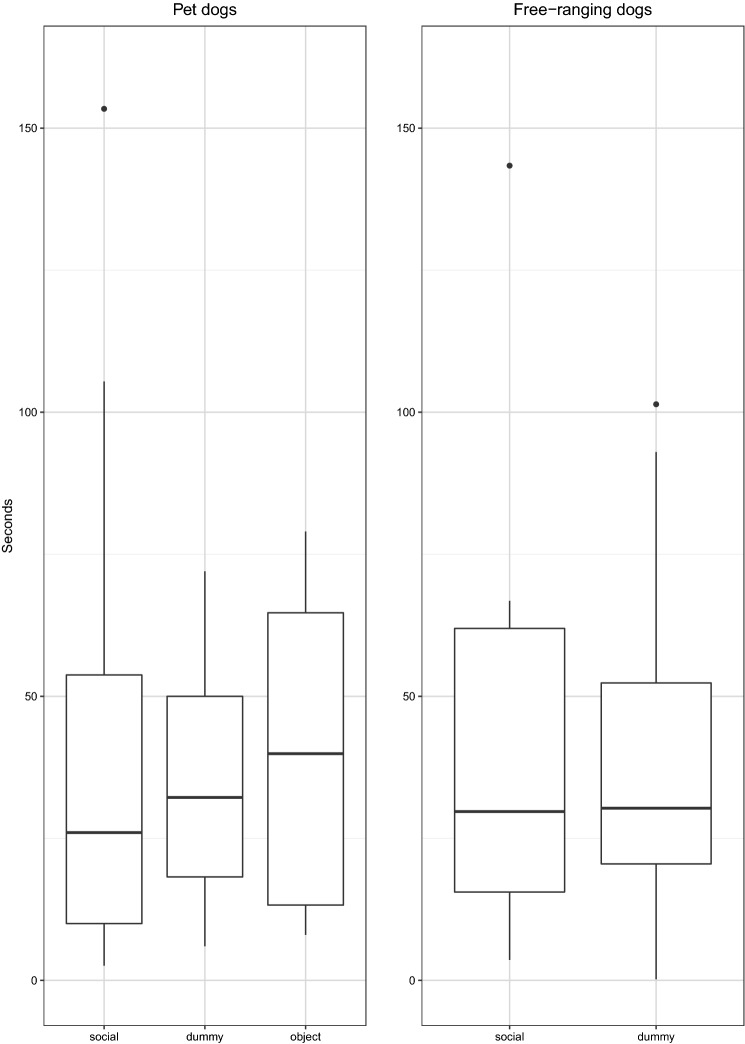
Latency to look back after interacting with the impossible bowl for pet dogs (*N* = 20) and free-ranging dogs (*N* = 26). One pet dog tested in the dummy condition, with a value of 332.2, is not shown in the graph

*Latency to look back after trying to solve the impossible bowl*. Both the mixed model analysis and the Bayes factor analysis indicated that there was no effect of condition on latency to look back in both Pd and FRd and there was no difference in latency to look back between Pd and FRd in the social condition (see Table 4; Fig. 3) (Online Resource 1).

**Table 4:** Differences in latency, summary of statistics

Differences in latency	Model	Comparisons full-null model/tests	Bayes factor (support for H1)	Bayes factor (support for H0)
Across conditions in Pd	Model-L1	$\chi_{2}^{2}$ = 2.98, *p* = 0.22	0.18	5.42
Across conditions in FRd	Model-L2	*t* = 0.01, *p* = 0.93	0.41	2.42
Between Pd and FRd (social condition)	Model-L3	*t* = − 0.32, *p* = 0.75	0.37	2.67

*Pd* pet dogs, *FRd* free-ranging dogs

*Frequency of looking back*. We found that the frequency of looking back after attempting the impossible bowl differed between conditions in Pd (model-F1) (comparisons between the full and the null model, likelihood ratio test: $\chi_{2}^{2}$ = 15.5, *p* < 0.001). Pd looked more frequently at the human than at the 'dummy' human and object (social object: *z* = 3.25, *p* = 0.001; social dummy: *z* = − 2.66, *p* = 0.007), with no difference between the frequency of looking at the ‘dummy’ human and at the object (dummy object: *z* = 1.2, *p* = 0.23) (Online Resource 1). In FRd, there were no difference between conditions (social and dummy) in the frequency of looking back after attempting the impossible bowl (model-F2) (*z* = 0.32, *p* = 0.74) (Online Resource 1). Furthermore, there was no difference in the frequency of looking back at the experimenter after attempting the impossible bowl between Pd and FRd (model-F3) (*z* = − 0.5, *p* = 0.56) (Online Resource 1). Finally, considering only the social condition in which the experimenter was present, Pd and FRd did not differ in the frequency of looking back when attempting either the possible or the impossible bowl (model-F4) (comparisons between the full and the null model, likelihood ratio test: $\chi_{2}^{2}$ = 0.87, *p* = 0.64) (Online Resource 1).

**Fig. 4:**
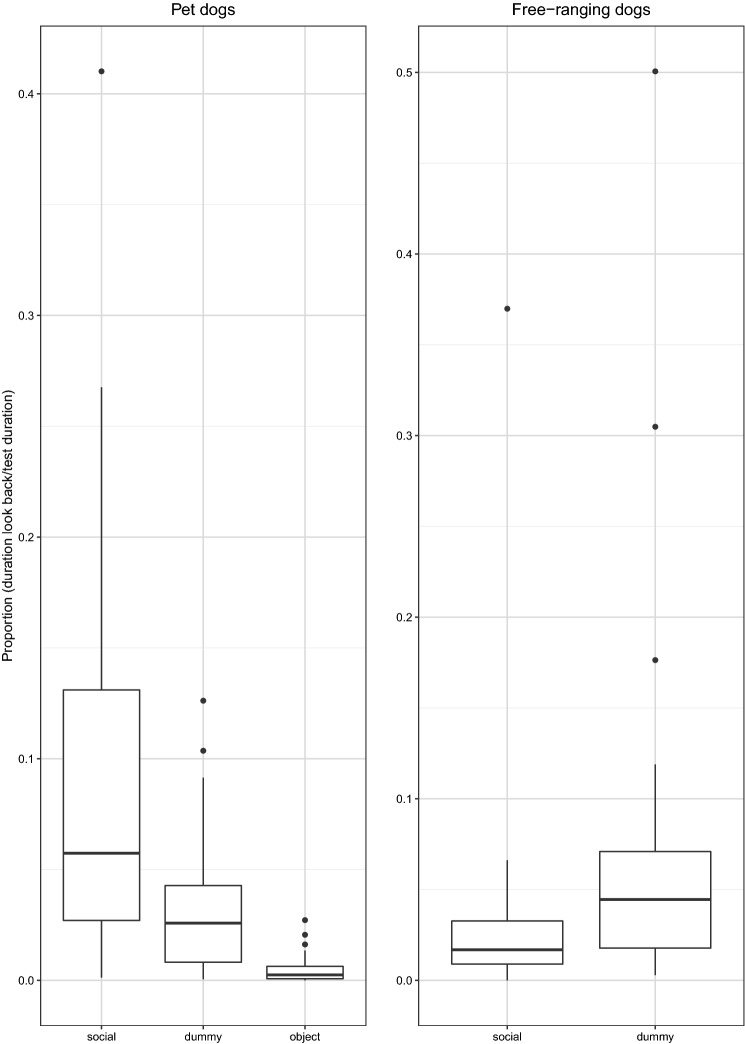
Proportion of time subjects looked back over the entire test in three test conditions (object, dummy, social) for pet dogs (*N* = 20) and in the two test conditions (dummy, social) for free-ranging dogs (*N* = 31)

**Fig. 5:**
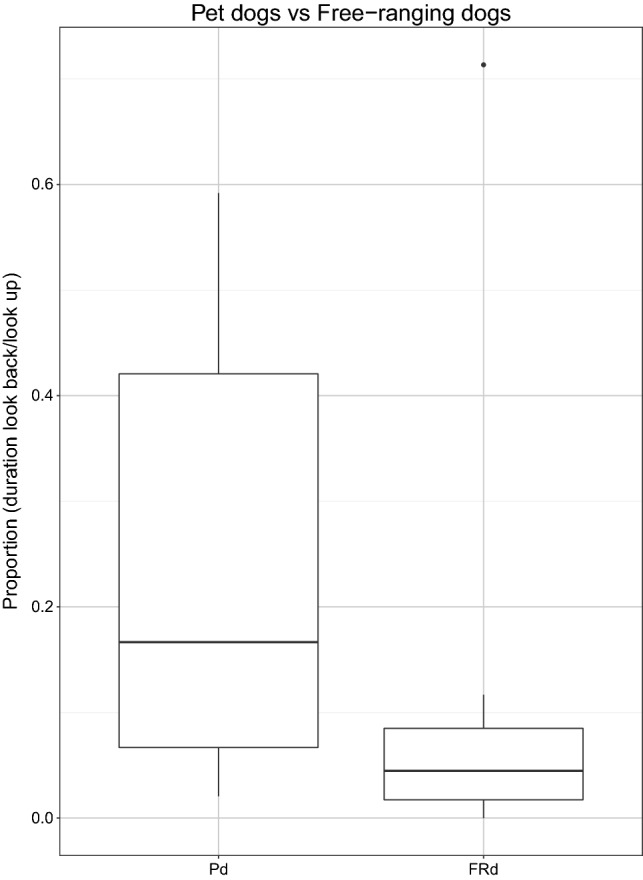
Time spent looking back as a proportion of time spent looking up (in the social condition) for pet dogs (*N* = 19) and free-ranging dogs (*N* = 14)

*Duration of looking back over the entire test.* We found that the duration of looking back differed between the three conditions in Pd (see Table 5). Pd looked longer at the human than at the ‘dummy’ human and object (social object: *z* = 4.8, *p* < 0.0001; social dummy: *z* = 3.4, *p* < 0.001) and longer at the ‘dummy’ human than at the object (dummy-object: *z* = − 2.39, *p* = 0.01). In contrast, no difference in the duration of looking back was found between the social and the dummy condition in FRd (see Table 5; Fig. 4). We found that Pd tended to look back at the experimenter for longer than did FRd (see Table 5; Fig. 5) (Online Resource 1).

**Table 5:** Differences in the overall duration of looking back, summary of statistics

Differences in duration of looking back	Model	Comparisons full-null model/tests	Bayes factor (support for H1)	Bayes factor (support for H0)
Across conditions in Pd	Model-DL1	$\chi_{2}^{2}$ = 21.75, *p* < 0.001	897.88	0.001
Across conditions in FRd	Model-DL2	*z* = − 0.8, *p* = 0.42	0.34	2.87
Between Pd and FRd (social condition)	Model-DL3	*z* = 1.87, *p* = 0.06	2.19	0.45

*Pd* pet dogs, *FRd* free-ranging dogs

**Fig. 6:**
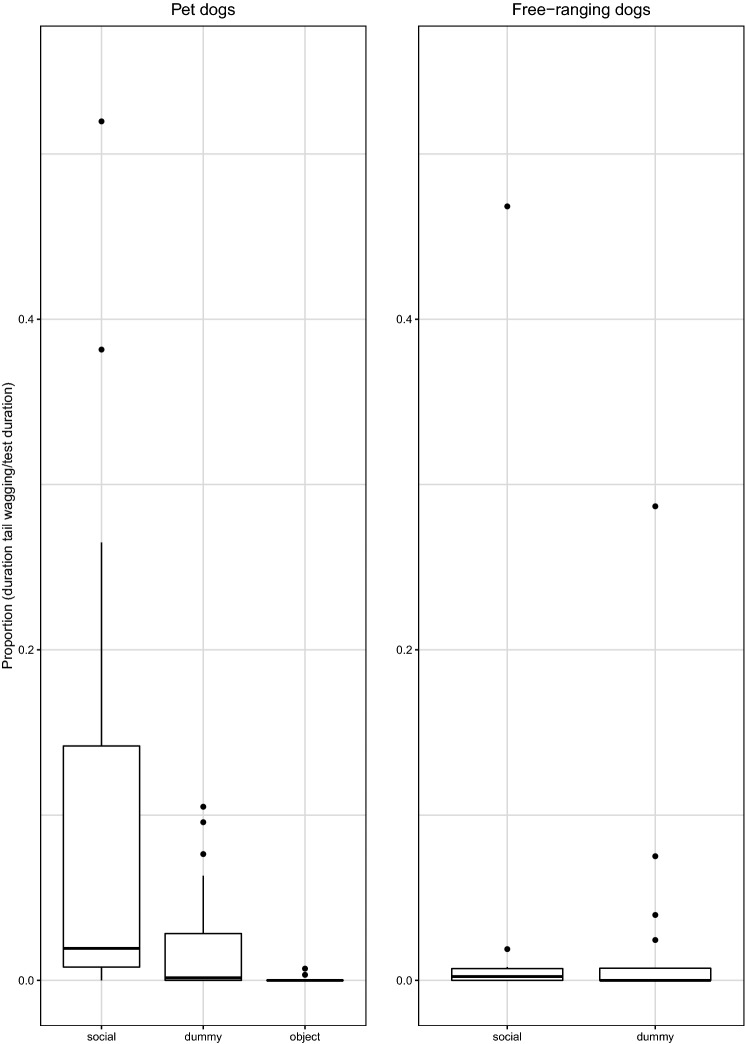
Proportion of time subjects wagged their tails over the entire test in three test conditions (dummy, object, social) for pet dogs (*N* = 20) and in the two test conditions (dummy, social) for free-ranging dogs (*N* = 31)

*Duration of tail wagging during the entire test*. The duration of tail wagging was positively correlated with the duration of looking back behaviour for both groups (Pearson’s correlation: Pd Cor. coeff = 0.83; *p* < 0.0001; FRd Cor. coeff = 0.78; *p* < 0.0001). We found that the duration of tail wagging differed between the three conditions in Pd (see Table 6). Pd performed tail wagging for a longer period in the social condition than in the ‘dummy’ human condition and in the object condition (social dummy: *z* = 3.68, *p* < 0.0001; social object: *z* = 4.14, *p* < 0.0001) and for longer in the ‘dummy’ human condition than the object condition (dummy-object: *z* = 2.39, *p* = 0.02) (see Table 6). Overall, there were no differences in the duration of tail wagging between the social and dummy condition in FRd (see Table 6). There were no differences between Pd and FRd in the duration of tail wagging in the social condition (see Table 6; Fig. 6) (Online Resource 1).

**Table 6:** Differences in duration of tail wagging, summary of statistics

Differences in duration of tail wagging	Model	Comparisons full-null model, likelihood ratio test	Bayes factor (support for H1)	Bayes factor (support for H0)
Across conditions in Pd	Model-W1	$\chi_{2}^{2}$ = 10.15, *p* = 0.006	2.7	0.37
Across conditions in FRd	Model-W2	*z* = − 0.03, *p* = 0.97	0.18	5.56
Between Pd and FRd (social condition)	Model-W3	*z* = 0.97, *p* = 0.32	0.44	2.23

*Pd* pet dogs, *FRd* free-ranging dogs

## Discussion

Overall, the results indicate that the looking back behaviour in an impossible task is not a problem-solving strategy (i.e. the dogs do not look back to ask for help) but is rather a consequence of giving up and looking at the most salient object in the environment (hypothesis 2a).

If looking back was a social strategy, then dogs should have tried to solve the task by themselves for less time when in the presence of a human that could help them, than in the absence of a human. But this was not the case. Furthermore, if looking back was a social strategy, then pet dogs, used to humans helping them in various ways, should have shown this pattern of results more strongly than free-ranging dogs that have never experienced human ‘help’. Contrary to this, we found that pet dogs and free-ranging dogs had a similar persistence in the social condition and non-social conditions, and we found no difference between free-ranging and pet dogs in their latency to look back at the human. Interestingly, dogs in this study showed a similar persistence (social condition: Pd mean 53.1 s; FRd mean 42.74 s) to different groups of dogs from a previous study (i.e. Indian free-ranging dogs: mean 60 s, captive dogs living in packs: mean 46.5 s). This suggests that persistence is rather constant across different dog populations in this test paradigm and independent of the human presence and the subject’s past experience (Marshall-Pescini et al. 2017). Furthermore, as suggested by Marshall-Pescini et al. (2017), the latency to look back in this testing paradigm seems to be mainly determined by subject’s persistence: the animals give up trying to solve the apparatus after a certain amount of time and then look around at the most salient object in their environment—either the experimenter or any other object that stands out. Considering the current results and the suggested link between latency and persistence, observed differences in latency to look back in previous studies (Hori et al. 2013; Passalacqua et al. 2011; Miklósi et al. 2003), might have been determined by differences in persistence between subjects (but see Konno et al. 2016; D’Aniello and Scandurra 2016) as has already been found in dogs and wolves (Rao et al. 2018; Marshall-Pescini et al. 2017). The previous studies mainly focused on latency, rarely analysing persistence which should instead be considered one of the main factors influencing subjects’ looking behaviour in similar testing paradigms.

One partial limitation of the study is that whereas pet dogs carried out all test conditions, in free-ranging dogs we were able to re-test only a subsample of dogs. However, it is important to note that the comparison between free-ranging dogs and pet dogs was carried out on this sub-population and only in the social condition, and in both populations the order of presentation was counterbalanced.

Interestingly, a few differences in the looking back behaviour between free-ranging and pet dogs did emerge. Whereas free-ranging dogs looked back as frequently (and for as long) at the human and at the ‘dummy’ human, pet dogs looked back more frequently (and for longer) at the human than at the control objects. The fact that free-ranging dogs looked for a similar duration at the human and at the ‘dummy’ human may suggest that free-ranging dogs were a bit fearful of the human shape (a novel object in their environment), or perhaps needed a bit more time to figure out what this stimuli was (video V1). Interestingly, based on their tail-wagging behaviour being of similar duration for both human and dummy human, it would appear that the dogs, at least to begin with, may have approached the dummy human in a ‘social’ manner (Quaranta et al. 2007). Pet dogs, on the other hand differentiated more between the social and control stimuli, looking and tail wagging more towards the experimenter, than the dummy human, while they also looked and tail wagged more towards the dummy than the cardboard. This gradient is interesting, since it would suggest they at least initially, treated the ‘Han Solo’ cut-out as more ‘human-like’ than the cardboard. These results may suggest that pet dogs are in general better at quickly discriminating between humans and other odd objects in their environment than free-ranging dogs. However, they do not support the idea of looking back as a problem-solving strategy: if looking back was a problem-solving strategy we would have expected pet dogs to look back more often specifically after attempting the impossible bowl (and not when manipulating the possible bowls), but this was not the case.

Finally, we found that in the social condition pet dogs tended to look longer (during the whole test) at the experimenter than did free-ranging dogs. This difference could not be attributed to free-ranging dogs being scared of humans since the tested population had a friendly attitude towards humans (they are not feral dogs that have ‘dishabituated’ to the presence of humans). These ‘village’ dog populations are commonly observed all over the world and have already been tested in different cognitive studies investigating dog–human interactions (Bhattacharjee et al. 2017a, b; 2018; Brubaker et al. 2017, 2019; Marshall-Pescini et al. 2017). Rather, we suggest that the greater duration of the looking behaviour in pet dogs, may simply be because they are more attracted to humans, potentially because they form stronger, more long-lasting bonds with humans than the free-ranging dogs in our population and/or because of their long history of associating humans (and potentially looking at humans) with food (Bentosela et al. 2008, 2009; D’Aniello and Scandurra 2016; Hall 2017).

The results of the previous studies on the impossible task also highlight the importance of previous reinforcement history. Trained dogs looked for longer at the human than untrained dogs (Marshall-Pescini et al. 2009; D’Aniello et al. 2015), and dogs trained for ‘agility’ (i.e. trained to specifically look at the human) looked for longer at the owner than untrained dogs and dogs trained for ‘search and rescue’ (Marshall-Pescini et al. 2009). Additionally, dogs living in kennels, with limited contact with humans, looked back for a shorter duration than untrained pet dogs (D’Aniello and Scandurra 2016). Finally, older dogs, which indeed have a longer experience of associating food with looking at humans, looked for longer at the human than did younger dogs (Hori et al. 2013; Passalacqua et al. 2011). It is worth noting that in some of these studies, the authors expected to find exactly the opposite results: the more experienced the dogs were, the less they should have looked back at the human if asking for help. Overall, pet dogs’ experience, whereby looking is likely to be both intentionally and inadvertently reinforced by humans, strongly affects looking patterns in such experimental settings, highlighting the importance of subjects’ previous ontogenic background.

In conclusion, our results show that dogs’ looking back behaviour in an impossible task which does not represent a social ‘help-seeking’ strategy. The latency of looking back is rather linked to the subject’s persistence, whereas the frequency and duration of looking back are rather linked to the salience of the stimuli presented, and potentially to the past reinforcement history of the study population. This behaviour has been widely over-interpreted and more caution should be exercised in future studies.

## Electronic supplementary material

Below is the link to the electronic supplementary material.

Supplementary file1 (PDF 512 kb)

Supplementary file2 (PDF 445 kb)

Supplementary file3 (XLSX 30 kb)

Supplementary file4 (PDF 531 kb)

Video: Why do dogs look back at the human in an impossible task? Looking back behaviour may be over-interpreted (MP4 426729 kb)
