# Supplementary material for: Why do dogs look back at the human in an impossible task? Looking back behaviour may be over-interpreted
Source: Anim Cogn. 2020 Feb 23;23(3):427–41. doi: 10.1007/s10071-020-01345-8 (PMC7181563; doi:10.1007/s10071-020-01345-8)
Supplement: Supplementary file 1 — Supplementary file1 (PDF 512 kb) [file 10071_2020_1345_MOESM1_ESM.pdf]

SUPPLEMENTARY MATERIALS

Why do dogs look back at the human in an impossible task? Looking back behaviour may be over-interpreted.

Martina Lazzaroni[1], Sarah Marshall-Pescini[1], Helena Manzenreiter[2], Sarah Gosch[2], Lucy Přibilová[2], Larissa Darc[1], Jim McGetrick[1], Friederike Range [1]
[1]Domestication Lab, Konrad Lorenz Institute of Ethology, University of Veterinary Medicine, Vienna, Austria
[2]Comparative Cognition, Messerli Research Institute, University of Veterinary Medicine, Vienna, Austria

Corresponding authors
E-mail: martina.lazzaroni@vetmeduni.at.ac
E-mail: friederike.range@vetmeduni.at.ac

The following are reported for all models: the estimates, together with standard errors, tests, confidence limits, as well as minimum and maximum of estimates derived after excluding individuals one at a time (model stability).

Table 1. Results of the model-P1: differences in persistence across conditions in pet dogs.

term	Estimate	SE	χ^2	p	lower Cl	upper Cl	min	max
(Intercept)	4.018	0.358	(1)	(1)	-0.366	0.913	3.824	4.241
condition (dummy) (2)	0.273	0.321	(1)	(1)	-0.978	0.325	-0.137	-0.395
condition (object) (2)	-0.328	0.327	(1)	(1)	-0.952	0.349	-0.481	-0.162
condition (social) (2)	-0.303	0.326	(1)	(1)	-0.342	0.079	-0.420	-0.097
test	-0.131	0.106	1.51	0.218	-0.366	0.913	-0.177	-0.093

(1) not indicated because of having a very limited interpretation
(2) condition 'alone' is the reference category

Table 2. Results of the model-P2: differences in persistence across conditions in free-ranging dogs.

term	Estimate	SE	χ^2	p	lower Cl	upper Cl	min	max
(Intercept)	4.401	0.711	(1)	(1)	2.941	5.866	4.069	4.889
condition (social) (2)	0.302	0.459	0.430	0.512	-0.639	1.255	0.073	0.523
test	-0.871	0.461	3.284	0.069	-1.824	0.077	-1.077	-0.644

(1) not indicated because of having a very limited interpretation
(2) condition 'alone' is the reference category

Table 3. Results of the model-P3: differences in persistence between pet dogs and free-ranging dogs in the social condition .

term	Estimate	SE	t	p	lower CI	upper CI	min	max
(Intercept)	3.233	0.339	(1)	(1)	2.538	3.927	2.945	3.406
group (pet dogs) (2)	0.151	0.441	0.342	0.735	- 0.750	1.052	-0.023	0.439

(1) not indicated because of having a very limited interpretation

(2) group 'free-ranging dogs' is the reference category

Table 4. Results of the model-L1: differences in latency to look back across conditions in pet dogs.

term	Estimate	SE	z	p	lower CI	upper CI
condition (dummy) (1)	-0.737	0.890	-0.83	0.410	-26.796	1.398
condition (social) (1)	-1.928	1.161	-1.66	0.097	-22.969	-0.989

(1) condition 'object' is the reference category

Table 5. Results of the model-L2: differences in latency to look back across conditions in free-ranging dogs.

term	Estimate	SE	t	p	lower CI	upper CI	min	max
(Intercept)	5.729	1.088	(1)	(1)	3.422	8.036	4.975	6.349
condition (social) (2)	0.133	1.459	0.091	0.929	-2.962	3.227	-0.487	0.887

(1) not indicated because of having a very limited interpretation

(2) condition 'dummy' is the reference category

Table 6. Results of the model-L3: differences in latency to look back between pet dogs and free-ranging dogs in the social condition.

term	Estimate	SE	t	p	lower CI	upper CI	min	max
(Intercept)	3.272	0.351	(1)	(1)	2.550	3.994	3.051	3.461
group (pet dogs) (2)	- 0.141	0.438	-0.322	0.75	- 1.041	0.759	- 0.329	0.080

(1) not indicated because of having a very limited interpretation

(2) group 'free-ranging dogs' is the reference category

Table 7. Results of the model-F1: differences in frequency of looking back across conditions in pet dogs.

term	Estimate	SE	z	p	lower CI	upper CI	min	max
(Intercept)	-3.146	0.466	(1)	(1)	-4.205	-2.335	-4.014	-2.959
condition(dummy) (2)	0.658	0.548	1.201(3)	0.229(3)	-0.363	1.835	0.405	1.565
condition (social) (2)	1.643	0.506	3.250(3)	0.001(3)	0.727	2.755	1.477	2.588

(1) not indicated because of having a very limited interpretation

(2) condition 'object' is the reference category

Table 8. Results of the model-F2: differences in frequency of looking back across conditions in free-ranging dogs.

term	Estimate	SE	z	p	lower CI	upper CI	min	max
(Intercept)	-1.408	0.336	(1)	(1)	-2.119	-0.787	-1.663	-1.293
condition (social) (2)	0.146	0.446	0.329	0.742	-0.722	1.039	-0.018	0.401

(1) not indicated because of having a very limited interpretation

(2) condition 'dummy' is the reference category

Table 9. Results of the model-F3: differences in frequency of looking back at the human between pet dogs and free-ranging dogs.

term	Estimate	SE	z	p	lower CI	upper CI	min	max
(Intercept)	-1.262	0.292	(1)	(1)	- 1.871	- 0.715	- 1.427	- 1.156
group (pet dogs) (2)	- 0.209	0.359	- 0.583	0.56	- 0.904	0.514	- 0.353	- 0.045

(1) not indicated because of having a very limited interpretation

(2) group 'free-ranging dogs' is the reference category

Table 10. Results of the model-F4: differences between pet dogs and free-ranging dogs in frequency of looking back at the human after attempting the possible or the impossible bowls.

term	Estimate	SE	z	p	lower CI	upper CI	min	max
(Intercept)	0.449	0.356	(1)	(1)	-0.215	1.218	0.294	0.608
group (pet dogs) (2)	0.187	0.449	0.416	0.678	-0.765	1.105	-0.013	0.482
poss-imp	-0.379	0.494	- 0.767	0.443	-1.411	0.668	-0.546	-0.085

(1) not indicated because of having a very limited interpretation

(2) group 'free-ranging dogs' is the reference category

Table 11. Results of the model-DL1: differences in duration of looking back across conditions in pet dogs.

term	Estimate	SE	z	p	lower CI	upper CI	min	max
(Intercept)	-3.116	0.215	(1)	(1)	- 3.536	- 2.695	- 3.296	- 3.049
condition (object) (2)	- 0.615	0.257	- 2.395	0.017	- 1.118	- 0.112	- 0.697	- 0.534
condition (social) (2)	0.800	0.235	3.404	<0.001	0.339	1.261	0.712	1.022

(1) not indicated because of having a very limited interpretation

(2) condition 'dummy' is the reference category

Table 12. Results of the model-DL2: differences in duration of looking back across conditions in free-ranging dogs.

term	Estimate	SE	z	p	lower CI	upper CI	min	max
(Intercept)	-2.196	0.304	(1)	(1)	-2.614	-1.779	-2.447	-2.142
condition (social) (2)	-0.346	0.430	-0.805	0.421	-0.936	0.244	-0.499	-0.268

(1) not indicated because of having a very limited interpretation

(2) condition 'dummy' is the reference category

Table 13. Results of the model-DL3: differences between pet dogs and free-ranging dogs in duration of looking back at the experimenter.

term	Estimate	SE	z	p	lower CI	upper CI	min	max
(Intercept)	-1.885	0.279	(1)	(1)	-2.432	-1.339	-2.176	-1.821
group (pet dogs) (2)	0.744	0.396	1.876	0.06	0.104	1.384	0.681	0.962

(1) not indicated because of having a very limited interpretation

(2) group 'free-ranging dogs' is the reference category

Table 14. Results of the model-W1: differences in duration of tail wagging across conditions in pet dogs.

Term	Estimate	SE	z	p	lower CI	upper CI	min	max
(Intercept)	-3.576	0.343	(1)	(1)	- 4.249	- 2.903	- 3.852	- 3.127
condition (object) (2)	- 0.723	0.308	- 2.349	0.018	-1.326	- 0.119	-0.890	- 0.277
condition (social) (2)	1.050	0.285	3.678	<0.001	0.490	1.609	- 0.511	1.328

(1) not indicated because of having a very limited interpretation

(2) condition 'dummy' is the reference category

Table 15. Results of the model-W2: differences in duration of tail wagging across conditions in free-ranging dogs.

Term	Estimate	SE	z	p	lower CI	upper CI	min	max
(Intercept)	-2.923	0.441	(1)	(1)	-3.399	-2.448	-3.279	-2.893
condition (social) (2)	-0.019	0.561	-0.033	0.97	-0.624	0.586	-0.215	0.082

(1) not indicated because of having a very limited interpretation

(2) condition 'dummy' is the reference category

Table 16. Results of the model-W3: differences between pet dogs and free-ranging dogs in duration of tail wagging at the experimenter.

Term	Estimate	SE	z	p	lower CI	upper CI	min	max
(Intercept)	-2.577	0.417	(1)	(1)	-3.159	-1.995	-2.867	-2.546
group (pet dogs) (2)	0.449	0.459	0.977	0.328	-0.193	1.092	0.393	0.617

(1) not indicated because of having a very limited interpretation

(2) group 'free-ranging dogs' is the reference category
