# Supplementary material for: Why do dogs look back at the human in an impossible task? Looking back behaviour may be over-interpreted
Source: Anim Cogn. 2020 Feb 23;23(3):427–41. doi: 10.1007/s10071-020-01345-8 (PMC7181563; doi:10.1007/s10071-020-01345-8)
Supplement: Supplementary file 2 — Supplementary file2 (PDF 445 kb) [file 10071_2020_1345_MOESM2_ESM.pdf]

SUPPLEMENTARY MATERIALS

Why do dogs look back at the human in an impossible task? Looking back behaviour may be over-interpreted.

Martina Lazzaroni[1], Sarah Marshall-Pescini[1], Helena Manzenreiter[2], Sarah Gosch[2], Lucy Přibilová[2], Larissa Darc[1], Jim McGetrick[1], Friederike Range [1]

[1]Domestication Lab, Konrad Lorenz Institute of Ethology, University of Veterinary Medicine, Vienna, Austria
[2]Comparative Cognition, Messerli Research Institute, University of Veterinary Medicine, Vienna, Austria

Corresponding authors
E-mail: martina.lazzaroni@vetmeduni.at.ac
E-mail: friederike.range@vetmeduni.at.ac

Additional analyses on test duration.

Since the test ended when the subject did not interact with the apparatus for more than five minutes, the test duration varied across subjects. To investigate differences in test duration between conditions we ran a generalized linear mixed model for Pd and a generalized linear model for FRd with test duration as the response variable, condition (social, dummy, object for Pd; social, dummy for FRd) as the explanatory factor and subject as a random factor (only for Pd). To investigate differences in test duration between Pd and FRd in the social condition we ran a generalized linear model with test duration as the response variable and group (Pd, FRd) as the explanatory factor. All models fitted the assumption of normally distributed residuals and were of moderate or good stability.

Overall there were no differences in the test duration across different conditions in Pd (comparisons between the full and the null model, likelihood ratio test: χ^2_2= 0.83, p=0.66). However, we found that the test duration differed between the two conditions in FRd (t= 2.92, p=0.006), where the social condition lasted significantly longer than the 'dummy' human condition. We found that the social condition tests lasted longer in Pd than in FRd (t=2.64, p=0.013) (Pd: mean 498.16 s, range 305-644.4 s; FRd: mean 411.04 s, range 319-472.6 s).

Following are reported for all models the estimates, together with standard errors, tests, confidence limits, as well as minimum and maximum of estimates derived after excluding individuals one at a time (model stability).

Table 1. Differences in test duration for social, dummy and object conditions in pet dogs.

term	Estimate	SE	χ^2	p	lower Cl	upper Cl	min	max
(Intercept)	499.44	45.699	(1)	(1)	408.39	590.48	462.87	521.04
condition (object) (2)	-52.020	64.628	(1)	(1)	-180.78	76.738	-96.579	-15.231
condition (social) (2)	-1.277	65.473	(1)	(1)	-131.72	129.16	-24.371	38.809

(1) not indicated because of having a very limited interpretation

(2) condition 'dummy' is the reference category

Table 2. Differences in test duration for social and dummy conditions in pet dogs.

term	Estimate	SE	t	p	lower Cl	upper Cl	min	max
(Intercept)	288.53	28.16	(1)	(1)	230.93	346.13	106.88	304.16
condition (social) (2)	122.51	41.91	2.92	0.006	36.805	208.22	106.88	134.09

(1) not indicated because of having a very limited interpretation

(2) condition 'dummy' is the reference category

Table 2. Differences in test duration for the social condition between pet dogs and free-ranging dogs.

term	Estimate	SE	t	p	lower Cl	upper Cl	min	max
(Intercept)	411.04	24.97	(1)	(1)	360.11	461.97	403.96	416.18
group (pet dogs) (2)	87.12	32.91	2.647	0.013	20.004	154.24	76.389	100.03

(1) not indicated because of having a very limited interpretation

(2) group 'free-ranging dogs' is the reference category
