# Supplementary material for: Why do dogs look back at the human in an impossible task? Looking back behaviour may be over-interpreted
Source: Anim Cogn. 2020 Feb 23;23(3):427–41. doi: 10.1007/s10071-020-01345-8 (PMC7181563; doi:10.1007/s10071-020-01345-8)
Supplement: Supplementary file 4 — Supplementary file4 (PDF 531 kb) [file 10071_2020_1345_MOESM4_ESM.pdf]

SUPPLEMENTARY MATERIALS

Why do dogs look back at the human in an impossible task? Looking back behaviour may be over-interpreted.

Martina Lazzaroni[1], Sarah Marshall-Pescini[1], Helena Manzenreiter[2], Sarah Gosch[2], Lucy Přibilová[2], Larissa Darc[1], Jim McGetrick[1], Friederike Range [1]

[1]Domestication Lab, Konrad Lorenz Institute of Ethology, University of Veterinary Medicine, Vienna, Austria

[2]Comparative Cognition, Messerli Research Institute, University of Veterinary Medicine, Vienna, Austria

Corresponding authors

E-mail: martina.lazzaroni@vetmeduni.at.ac

E-mail: friederike.range@vetmeduni.at.ac

Summary tables of data used in the analyses.

Group	Condition	Persistence (sec)	Latency of looking back (sec)	Duration of looking back (sec)	Duration of tail wagging (sec)	Test duration (sec)
pet dogs	social	53.1 ± 77.44	38.31 ± 39.81	46.93 ± 47.74	43.27 ± 63.34	498.16 ± 112.04
	dummy	94.91 ± 117.24	49.73 ± 73.33	15.54 ± 21.17	7.83 ± 13.28	499.44 ± 271.5
	object	60 ± 97.65	39.78 ± 27.03	2.37 ± 3.12	0.29 ± 0.939	447.42 ± 209.9
free-ranging dogs	asocial	100.49 ± 186.89	*	*	*	459.52 ± 299.8
	social	48.38 ± 56.2	42.7 ± 42.09	18.58 ± 37.55	14.76 ± 48.94	411.04 ± 58.60

Table 1. Mean and standard deviation of durations, in pet dogs and free-ranging dogs.

Group	Condition	Frequency of looking back imp (1)	Frequency of looking back poss (2)	Frequency of attempting the imp	Frequency of look up poss (2)	Frequency of look up imp (1)
pet dogs	social	1.47 ± 1.26	0.26 ± 0.47	7.89 ± 3.43	0.6 ± 0.63	2.93 ± 1.67
	dummy	0.65 ± 0.74	*	8.65 ± 6.58	*	*
	object	0.26 ± 0.73	*	6.26 ± 4.28	*	*
free-ranging dogs	social	1.07 ± 0.83	0.85 ± 0.77	4.86 ± 3.06	1.57 ± 1.74	3.14 ± 2.21
	dummy	0.78 ± 0.67	*	4 ± 3.23	*	*

Table 2. Mean and standard deviation of frequencies (number of times a behaviour was performed), in pet dogs and free-ranging dogs; imp: impossible bowl, poss: possible bowls. (1) number of times the subjects looked back or looked up after attempting the impossible bowl; (2) number of times the subjects looked back or looked up after attempting the possible bowls (note that looking back in this case is considered only if it happened in the two second period after the subject stopped interacting with the bowl. Additionally, note that after interacting with the possible bowls subjects where busy eating the food on the ground while this did not happen after interacting with the impossible bowl).
